# A Case of COVID-19-Related Thrombocytopenia and Leukopenia in an Adolescent with Mild Symptoms

**DOI:** 10.3390/children8060509

**Published:** 2021-06-16

**Authors:** Lydia Kossiva, Athanasios Thirios, Eleni Panagouli, Alexandros Panos, Stavroula Lampidi, Flora Bacopoulou, Maria Tsolia, Artemis Tsitsika

**Affiliations:** 1 2nd Department of Pediatrics, “P. & A. Kyriakou” Children’s Hospital, School of Medicine, National and Kapodistrian University of Athens, 115 27 Athens, Greece; lydiakossiva@hotmail.com (L.K.); athirios@med.uoa.gr (A.T.); elenpana@med.uoa.gr (E.P.); alxpanos@gmail.com (A.P.); stavroul.lab@gmail.com (S.L.); mariantsolia@gmail.com (M.T.); 2 Center for Adolescent Medicine and UNESCO Chair Adolescent Health Care, First Department of Pediatrics, “Agia Sophia” Children’s Hospital, School of Medicine, National and Kapodistrian University of Athens, 115 27 Athens, Greece; bacopouf@hotmail.com

**Keywords:** COVID-19, leucopenia, platelet count, bone marrow, myelodysplastic syndromes

## Abstract

Since the beginning of the COVID-19 pandemic, there have been numerous reports and reviews on the complications caused by the disease, analyzing the acute and chronic consequences. The main symptoms of SARS-CoV-2 are dry cough, fever, and fatigue. COVID-19 appears to affect all systems, including renal, cardiovascular, circulatory, and respiratory systems, causing chronic obstructive pulmonary disease. We report on a 14-year-old male adolescent, who presented with thrombocytopenia (platelet count 92 × 10^9^ /L) and leukopenia (white blood count 4.2 × 10^3^ /μL) that was observed two months ago. Ten days before the first blood test, a viral infection with nasal congestion and runny nose was reported, without other accompanying symptoms. Viral antibodies screening revealed positivity for all the three specific COVID-19 antibodies. Further haematological evaluation with bone marrow aspiration revealed non-specific dysplastic features of the red cell and megakaryocyte progenitors. Although haematological alterations due to COVID-19 infection are available from adult patients’ reports, the effect of COVID-19 infection in the pediatric population is underestimated and this is the first case with such haematological involvement. Noteworthy, in the current case, the impact of the COVID-19 infection was not related to the severity of the disease, as the symptoms were mild. In similar cases, bone marrow aspiration would not be performed as a part of routine work-up. Thus, it is important when evaluating pediatric patients with COVID-19 infection to search and report those alterations in order to better understand the impact and the spectrum of clinical manifestations of the specific viral infection in children and adolescents.

## 1. Introduction

SARS-CoV-2 belongs to the genus β of the coronavirus family. It is a new coronavirus related to severe acute respiratory syndrome (SARS-CoV) and the cause of the current pandemic. SARS-CoV-2 shares the same clinical symptoms with the other coronaviruses but it has the ability to bind to angiotensin-converting enzyme 2 (ACE-2) receptors 10–20 times that of other coronaviruses [1]. Since the beginning of the COVID-19 pandemic, there have been numerous reports and reviews on the complications caused by the disease, analyzing the acute and chronic consequences that have arisen. The main symptoms of SARS-CoV-2 are dry cough, fever, and fatigue. COVID-19 appears to affect all systems, including renal, cardiovascular, circulatory, and of course, respiratory, causing chronic obstructive pulmonary disease [2].

A severe form of the SARS-CoV-2 disease in adult patients is a hyperinflammatory condition that affects all systems and may lead to multi-organ failure and death. Among pediatric COVID-19–infected patients, a small percentage develops a severe entity termed Multisystem Inflammatory Syndrome in Children (MIS-C), which is a hyper-inflammatory process due to viral infection and progresses to multiple organ failure, resembling the same catastrophic inflammatory reaction observed in adults. Elevated inflammatory biomarkers are included in the diagnostic criteria for MIS-C [3]. Laboratory abnormalities in children with MIS-C more closely represent those seen in adults, with lymphopenia, and elevations of inflammatory biomarkers, D-dimer, cardiac troponin, and natriuretic peptides being commonly reported findings [4,5,6]. The most common hematological findings in patients with COVID-19 include reduced lymphocyte count, while the platelet count differs according to the severity of the disease as well as the total number of the white blood cell count [7,8,9]. Severe lymphopenia was linked to more severe disease compared to milder lymphopenia, although leukocytosis was referred as an indication of severe inflammation process [10]. 

Immune thrombocytopenia (ITP) is defined as a platelet count < 100  ×  10^9^/L. Petechiae or purpuric rashes are often present. ITP could be either primary or secondary. The most frequent causes of secondary ITP are several viral infections, such as varicella zoster virus, cytomegalovirus, hepatitis B/C viruses, or human immunodeficiency virus and, most recently, SARS-CoV-2 [11]. The majority of ITP cases, secondary to COVID-19, were reported three or four weeks after the onset of the illness in adult patients, while only a few reports on pediatric patients reported thrombocytopenia during the acute or the convalescent phase of the disease [12]. Changes of the red blood cell count during COVID-19 infection are reported in a small percentage of patients. In detail, lower hemoglobin levels along with a decrease of the mean corpuscular volume (MCV) are the main findings in some cases [13,14]. Only a handful of studies of the pediatric COVID-19 patients are available as far as hematological alterations are concerned.

We report on a case of secondary ITP along with borderline leukopenia and non- specific dysplastic features of the bone marrow, in a previous healthy 14-year-old male adolescent after a mildly symptomatic COVID-19 infection. Diagnosis of such cases is of high importance for the better understanding of the spectrum of COVID-19–infection consequences.

## 2. Case Presentation

A 14-year-old male adolescent attended the Adolescent Health Unit due to thrombocytopenia and leukopenia that was observed two months ago during an investigation for acute abdominal pain. A viral infection with nasal congestion and runny nose was reported ten days before the manifestation of the abdominal pain and the first haematological evaluation with full blood count (FBC: Hb 11.4 gr/dL (14–18), MCV 68 fL (79–98), WBC 4.2 cells/μL (4.0–10.0), PLT 92 × 10^9^/L).

The patient was reported previously healthy, with a history of tonsillectomy and adenoidectomy in infancy. He was the second child of non-consanguineous parents, born full-term with a normal perinatal period. He was fully vaccinated for his age, with normal weight and height (body mass: 73.5 kg, 90th percentile—height: 184 cm, <97 percentile—BMI: 21.7 kg/m^2^, 50 percentile).

According to the family history, his mother was completely healthy, while the father reported bronchial asthma, paroxysmal atrial fibrillation, and thyroidectomy nine years ago due to papillary thyroid cancer. No autoimmune diseases were mentioned in the family.

On admission in the Adolescent Health Unit, the adolescent was in excellent condition, with normal vital signs for his age (BP: 127/60 mmHg, HR: 78/min). The clinical examination revealed only a few petechiae on the back and the left clavicle. He underwent a thorough laboratory evaluation with complete blood count, biochemical markers, and extended viral and autoimmune panel screening. Thyroid function was also evaluated. The abdominal ultrasound and chest X-ray were both normal. He was tested negative for acute COVID-19 (PCR SARS-CoV-2) infection using a molecular (PCR) test, while the antibody titer against COVID-19 was sent and positive IgA, IgG, and IgM antibodies against COVID-19 were found (Table 1).

The alterations of two blood cell lines (leukopenia and thrombocytopenia) along with microcytosis (2+), hypochromy (1+), poikilocytosis (1+) of the red cell line and the age of the patient dictated further haematological and immunological evaluation. The bone marrow aspiration revealed that megakaryocytes were still present in normal numbers and producing platelets, while a small percentage of megakaryocyte progenitors displayed dysplastic features (micromegakaryocytes) along with mild non-specific dysplastic features of the erythrocyte progenitors. A small degree of non-specific hemophagocytosis was also present. A detailed evaluation towards myelodysplastic syndromes was performed with bone marrow karyotype, which was normal (46, XY), and fluorescent in situ hybridization (FISH) of the bone marrow, which was negative for monosomy 7/7q31del, 17, 5/5q31del, trisomy 8, and loss of p53. Fanconi anemia was excluded based on a negative chromosomal fragility screening test. A detailed and immunological work-up with immunoglobulin levels (IgG, IgA, IgM), immunophenotype of the peripheral lymphocyte subpopulations, C3, C4, and anti-nuclear and anti-ds DNA antibodies was performed and no indication of either immunodeficiency or autoimmune disease was found. The viral screening revealed positivity for all three specific COVID-19 antibodies (Table 1). The most possible cause of the ITP manifestation along with leukopenia and bone marrow dysplasia in our patient was considered to be a previous infection by SARS-CoV-2, probably the viral infection mentioned a few weeks ago. He did not receive any treatment and was discharged in excellent clinical condition after the completion of the clinical and laboratory evaluation. During follow-up, the adolescent remained asymptomatic and the platelet count was constantly rising while the white blood cell counts were still marginally low. Three months later (five months from the initial FBC) he is still leukopenic with borderline platelet number (Table 2).

## 3. Discussion

Our patient, even barely symptomatic at the beginning and without overt COVID-19 infection, exhibited thrombocytopenia, leukopenia, marginal lymphopenia, and non-specific dysplastic features in bone marrow aspiration. Based on the existing literature, only a few reports are available on haematological alterations due to COVID-19 infection, especially in children. This may be due to the fact that children are less vulnerable to severe disease, needing hospitalization, or their immature immune system reacts in a different way compared to adults. According to reports from adult patients with SARS-CoV-2, leukocytosis, lymphopenia, and various degrees of thrombocytopenia were linked to severe disease. Immune thrombocytopenia (ITP) has been associated with COVID-19 and seems to be an important risk factor for increased morbidity and mortality in those patients [8,11].

Bhattacharjee et al. have performed a systematic review concerning the clinical profile and outcomes of secondary ITP to COVID-19. In a total of 45 cases, the 71% were adults, aged over >50 years, while the 75% had moderate-to-severe COVID-19 and only 7% were asymptomatic. In 21%, ITP was observed about three weeks after the first diagnosis of COVID-19 and in post-recovery period. Only one associated case of death was recorded, due to intracranial hemorrhage and one case of secondary Evans syndrome [11]. Pediatric patients seem to be the minority, as only three cases (7%) of secondary ITP have been described. All the cases refer to children over 10 years old, two girls and one 16-year-old boy [15,16,17]. In two cases, low platelet count was observed after investigating a rash without other symptoms. Both children reported a mild illness before 3 to 4 weeks, the one was linked to COVID-19 and the other one was tested positive [15,17]. The third patient had an ongoing severe COVID-19 infection [16]. All of them received some kind of treatment for the ITP. Another paper from Tiwari et al. reported that 18.2% of pediatric COVID-19 cases exhibited thrombocytopenia, while in a meta-analysis paper from adult patients, the low platelet count was positively associated with the severity and the mortality of the disease [18]. Our patient had a negative COVID-19 PCR at the time of admission and recovered without any treatment. We might hypothesize that the lack of severe symptoms could be the reason for the mild thrombocytopenia. This is not the usual degree of post-viral thrombocytopenia attributed to other viruses, but in COVID-19, things might be different. The lower proportion of thrombocytopenia reported in children may be due to the milder clinical course and better prognosis of the disease compared to the adult COVID-19 patients.

Possible mechanisms suggesting the pathogenesis of ITP in COVID-19 disease include the direct infection of bone marrow cells [19]. It is speculated that SARS-CoV-2 decreases primary platelet formation through the inhibition of hematopoiesis in the bone marrow [20]. In detail, the bone marrow may be directly affected by the COVID-19 virus itself through specific receptors (CD13) on the progenitor hematopoietic cells, resulting in decreased platelet formation. Additionally, the virus may produce antibodies that cross-react with specific antigens on the platelet surface, through molecular mimicry, resulting in increased platelet destruction mainly from the macrophages of the spleen [20]. It is also suggested that, following the cytokine storm caused by SARS-CoV-2 infection, primary production of platelets from megakaryocytes decreases [21]. Finally, in overt disease, the increased platelet consumption due to aggregation and microthrombi formation, because of damaged pulmonary endothelial cells, enhances the platelet consumption [20]. The lower proportion of thrombocytopenia reported in children may be due to the milder clinical course and better prognosis of the disease compared to the adult COVID-19 patients.

Among other hemocytometry alterations, the presence of lymphopenia is a well-recognized finding linked to COVID-19, while leukocytosis is reported as a risk-marker in cases with hyperinflammation [22]. In most viruses, a lymphocytosis is present as a result of infection, while in SARS-CoV-2, infection lymphocyte depletion was noted. Several possible mechanisms for that have been suggested [1,23,24,25]. In detail, a lower % of CD4+ and CD8+ T-lymphocyte subpopulations have been described in patients with symptomatic COVID-19 infection. Lymphopenia associated with a low percentage of CD4+ and CD8+ T-lymphocytes returned to normal upon viral clearance [26]. According to a previous report from Lu et al., among 171 children with symptomatic COVID-19 infection, only six (3.5%) exhibited lymphopenia, even though in a meta-analysis from Henry et al., the aforementioned finding was much less common [27,28]. In a previous report from Qiu H et al., within a study group of 36 children with mild to moderate disease, 31% had low lymphocyte numbers and 19% had leukopenia [29]. In another report among 50 children with COVID-19 infection, 20% exhibited lymphopenia and 8% had an elevated number of lymphocytes [30]. Our patient had CD4+ and CD8+ T-lymphocyte subpopulations within normal range for his age but he exhibited a decrease of the total number of leukocytes and a marginally normal absolute number of neutrophils (1533/μL) (Table 1 and Table 2).

The presence of leukopenia has been reported in COVID-19 patients. Du et al., referred to severe leukopenia and lymphopenia in symptomatic rather than asymptomatic cases. A different response of the immature immune system in the younger age patients along with the limited data from the pediatric population could explain those findings [24,25]. Yarali et al. reported lymphopenia, neutropenia, and neutrophilia in 30%, 23.3%, and 13.3%, respectively, of the pediatric patients, despite the normal count of the leukocytes [24]. Other studies did not show the same alterations as far as the total number of white blood cells, lymphocytes, and neutrophils is concerned, as even in severe cases, the numbers of the aforementioned cell populations were within normal reference range [31]. A review paper from Kosmeri et al. concluded that the majority of children with COVID-19 infection exhibited normal WBC count and lymphopenia was not as frequently, as in adults, observed, possibly due to milder manifestation of the disease in younger ones [32]. Prognostic factors of the severity of the disease may be established based on the combination of several haematological parameters, such as the lymphocyte to neutrophil ratio, as reported in reports from adult patients [33]. The value of those prognostic factors is the prediction of critical illness in an early stage of the COVID-19 infection. Interestingly, our patient, even though mildly symptomatic, exhibited leukopenia, which was stable during follow-up. This is in accordance with a systematic review from Ma X et al. in which, among 486 hospitalized children, 21% had leukopenia and most of them had mild disease [34].

As for the red cell line, our patient exhibited mild anemia with low mean corpuscular volume (MCV). He did not have a history of thalassemia trait and his ferritin levels were within normal range (Table 1). In a large study from China among 244 pediatric patients, 193 had symptomatic COVID-19 infection and significantly lower levels of hemoglobin were found [14]. A lower level of hemoglobin has been reported along with a decrease in MCV, while those findings were more consistent in severely ill adult patients [35,36]. Infection by SARS-CoV-2 may directly affect the red cell precursors of the bone marrow either directly through ACE-2 receptors, leading to early apoptosis, or by inhibiting hematopoiesis though cytokine action in severely ill patients due to inflammatory process. A study from Yuan X et al. reported that critically ill patients had higher levels of IL-6, which is a well-recognized cytokine that affects hematopoiesis. Another possible mechanism of anemia may be due to hemophagocytosis of the red blood cells within the marrow or other sites such as lymph nodes and the spleen. The last mechanism is seen in severe cases and it is linked to high morbidity and mortality [1]. A paper from Liu and Li proposed certain SARS-CoV-2 proteins may interfere with erythropoiesis via attack of the beta chain of hemoglobin, resulting in a decrease of its level [37]. A reduction in hemoglobin could aggravate the respiratory distress syndrome in severely ill patients. In our case, there was no specific hemophagocytosis in the bone marrow and the rest of the diagnostic criteria (lipid profile, ferritin, coagulation panel) were within normal ranges and most of all our patients were asymptomatic and in excellent clinical condition. The non-specific dysplastic features of the red-cell progenitors were characterized as an asynchronous maturation process. Taking into account that the adolescent had absolutely normal complete blood counts in the past, we may hypothesize that COVID-19 infection, even with mild symptoms, might interfere with the normal hematopoiesis, leading to various degrees of anemia and microcytosis, but the exact mechanism of anemia is still obscure.

In our case, the presence of leukopenia with mild lymphopenia, along with thrombocytopenia and bone marrow non-specific dysplastic features of the red and megakaryocyte cell lines in an adolescent without previous hematological problems and in good clinical condition, is reported for the first time. It is noteworthy that three months after the first laboratory evaluation, the boy still has leukopenia and borderline number of platelets (Table 2). At this time, a repeat of the immunophenotype of lymphocyte subpopulations of the peripheral blood was within normal limits for his age. The fact that a rather mild COVID-19 infection caused numerical alterations in two cell lines (platelets and white blood cells) and quantitative and qualitative changes of the red blood cells (mild anemia and microcytosis) should be added to the spectrum of COVID-19 manifestations. It seems that some individuals may be more prone to hematological changes due to COVID-19 infection even if they exhibited mild clinical symptoms. As time passes, new COVID-19–related consequences will be added to its spectrum of manifestations. In our case, we may hypothesize that the mild viral infection, probably due to COVID-19 even though the patient was barely symptomatic, was responsible for alterations in all three cell lines of the bone marrow.

Concerning pediatric patients, we may underline that the incidence is underestimated due to the asymptomatic cases of COVID-19 and medical professionals must be aware and include SARS-CoV-2 infection within the panel of viral investigation of any type of haematological newly diagnosed pediatric cases. Even though lymphopenia and ITP are the most commonly reported haematological alterations until now, other haematological manifestations could be linked to COVID-19 infection in the future. Only limited data of the pediatric patients are available and the haematological alterations may be underestimated, as bone marrow aspiration is not a part of routine work-up especially in mild symptomatic cases. Thus, it is important to search and report those alterations during the COVID-19 pandemic as some cases may lead to severe, even life-threating sequelae. Additionally, a prognostic score based on classical hemocytometric parameters in pediatric patients may help in early identification of patients under risk for severe disease.

## Figures and Tables

**Table 1:** Initial laboratory evaluation.

**Immunoglobulings**	**Normal Values (mg/dL)**
IgG	1150 (863–1847)
IgA	128 (70–400)
IgM	100 (40–289)
C3	116 (90–180)
C4	16 (13–75)
**Virology panel:**	
Human Herpes Virus 6 (HHV6)	IgM(-), IgG(-)
Mycoplasma pneumoniae	IgM(-), IgG(-)
Herpes Simplex 1,2 (HSV1,2)	IgM(-), IgG(-)
Adenovirus	IgM(-), IgG(+)
Parvo B19	IgM(-), IgG(-)
Anti HBsAg	(-)
Anti-HBs	(+)
Anti-HCV	(-)
Human Immunodeficiency virus (HIV)	(-)
Toxoplasma gondii	IgM(-), IgG(-)
Cytomegalovirus (CMV)	IgM(-), IgG(-)
Epstein -Barr virus (EBV)	IgM(-), IgG(+)
SARs-COV-2 (PCR)	negative
SARs-COV-2 abs * IgA IgG IgM	POSITIVE 2.48 (negative < 0.8) 1.32 (negative < 0.8) 1.34 (negative < 0.28)
Ferritin	32.7 ng/ml (4.6–274)
B12	652 pg/ml (187–883)
TSH	1.27 mIU/ml (0.35–4.94)
Bleeding screening panel: (PT, aPTT, INR, Fibrinogen, D-dimers)	normal
Direct anti-globulin test: negative	negative
Immunophenotype of lymphocyte subpopulation of peripheral blood:	(CD3+) 72.1% (CD4+) 40.5% (CD8+)31.8%

* Enzyme linked Immunosorbent Assay (ELISA).

**Table 2:** Full blood count (FBC) on admission, discharge, and 3 months later.

Laboratory Tests	Admission to the Adolescent Health Unit	Admission to the Adolescent Health Unit	3 Months Later (Five Months from the Initial FBC)
WBC (/μL)	3.400	3900	4100
	PMN:45.3% (absolute number: 1533) LYM: 47.7% (absolute number: 1621) MONO: 4.9%	PMN: 44% (absolute number: 1716) LYM: 46%, (absolute number: 1794) MONO: 6%	PMN: 39.3% (absolute number: 1600) LYM: 53.1%, (absolute number: 2200) MONO: 5.4%
Hb (g/dL)	12.3	11.6	12.1
MCV (fL)	64	65.2	67.3
PLT(/μL)	86,000—platelet anisocytosis	130,000	112,000

WBC: white blood cells, Hb: hemoglobin, PLT: platelets, PMN: polymorphonuclear leukocytes, LYM: lymphocytes, MCV: mean corpuscular volume, MONO: monocytes.

## Data Availability

The datasets used in this study are available from the corresponding author.
